# User Experience, Actual Use, and Effectiveness of an Information Communication Technology-Supported Home Exercise Program for Pre-Frail Older Adults

**DOI:** 10.3389/fmed.2017.00208

**Published:** 2017-11-27

**Authors:** Marit Dekker-van Weering, Stephanie Jansen-Kosterink, Sanne Frazer, Miriam Vollenbroek-Hutten

**Affiliations:** ^1^Telemedicine Group, Roessingh Research and Development, Enschede, Netherlands; ^2^Biomedical Signals and Systems Group, Faculty of Electrical Engineering, Mathematics and Computer Science, University of Twente, Enschede, Netherlands; ^3^ZiekenhuisGroep Twente (ZGT), Scientific Office ZGT Academy, Almelo, Netherlands

**Keywords:** older adults, pre-frail, e-health, active aging, functional decline

## Abstract

**Objective:**

The main objective of this study was to investigate the use and user experience of an Information Communication Technology-supported home exercise program when offered for independent use to pre-frail older adults. Our secondary aim was to explore whether the program improved quality of life and health status compared to a control group.

**Methods:**

A cohort multiple randomized controlled trail is being performed. Physically pre-frail older adults (65–75 years) living independently at home were included and randomly assigned to a control group or an intervention group. The intervention group received a home exercise program (strength, balance, and flexibility exercises) for a minimal duration of 12 weeks. The control group received usual care. Primary outcomes were: use of the intervention (frequency and duration), adherence to a 3-day exercise protocol and user experience [System Usability Scale (SUS); rating 1–10]. Secondary outcomes were quality of life measured with the SF12 (Physical Component Scale and Mental Component Scale) and health status (EQ-5D), assessed before the study starts and after 12 weeks of exercising.

**Results:**

Thirty-seven independently living older adults participated in the study. Sixteen participants were allocated to the intervention group and 21 to the control group. The average score on the SUS was 84.2 (±13.3), almost reaching an excellent score. Participants rated the intervention with an 8.5. Eighty percent of the participants finished the 12 week exercise protocol. The adherence to the 3-day exercise protocol was 68%. Participants in the intervention group trained on average 2.2 times (±1.3) each week. The mean duration of login for each exercise session was 24 min. The Mental Component Scale of the SF12 was significantly higher in the intervention group compared to the control group. A trend was seen in the change over time in the health status between groups.

**Conclusions:**

This study provides evidence that a home-based exercise program is easy to use and has potential in improving quality of life and health status of pre-frail older adults who live at home. However, further refinement of the program is required to improve adherence and maximize the benefits and potential of exercising in the home environment.

**Trial Registration:**

Unique Identifier: NTR5304. URL: http://www.trialregister.nl/trialreg/admin/rctview.asp?TC=5304.

## Introduction

The number of older adults in our society rises (1) and health care professionals, including physiotherapists, are increasingly confronted with frail older adults (2). Frailty is an important predisposition for falls and associated with adverse health conditions (3). It is a major concern due to its high impact on affected individuals, their families, the health care system, and the society. As such, frailty should be treated in order to prevent the human and economic burden. The American College of Sports Medicine’s position stand recommend that exercise prescription for frail people is more beneficial than any other intervention and that resistance and/or balance training should precede the aerobic training for this population (4). However, to respond to demographical changes and the economic impact of frailty, it is of utmost importance to prevent frailty and by this focus on pre-frail older adults. Pre-frailty is considered to be an intermediate stage between non-frail and frail. Fried showed that pre-frail individuals have more than twice the risk of becoming frail compared with non-frail people (5). This highlights the importance of identifying and treating individuals in the pre-frail state before they become frail.

A recent systematic review showed that physical exercise interventions are beneficial for frail older adults in terms of functionality and activities of daily living and can delay progression of functional impairment or disability (6). Of the 19 interventions included, 15 were facility-based and only 4 were home-based interventions. Of those home-based exercise programs, the involvement and time investments of physiotherapists is very high (home visits) and limiting the self-management of the older adult. This indicates that home-based exercising is still quite a new field for (pre) frail older adults, especially as a self-management program. Nowadays, there is increasing interest in the role of “self-management” interventions to support the management of long-term conditions in health service settings. Self-management support has potential to improve the efficiency of health services by reducing other forms of utilization (such as primary care or hospital use) (7). As such, home-based self-management exercising might have more potential in reducing health care costs related to demographic changes compared to facility-based exercising. In addition, home-based self-management programs offer great potential for older adults as many older adults are reluctant to or unable to attend group exercise classes (8).

One widely used home-based exercise intervention is the Otago Exercise Programme (OEP) which is a cost-effective home-based individually tailored fall prevention program delivered by physiotherapists (9–11). The program has shown to improve participants’ strength and balance evaluated in older adults aged 65–97 years old (12, 13). However, the impact of this home-based exercise program in pre-frail older adults is unknown. As such, we developed an Information Communication Technology (ICT)-supported self-management exercise program based on OEP to prevent functional decline in a high-risk group of pre-frail older adults who live at home.

A major challenge that remains in this is appropriate use of such new technologies by older adults in their daily lives as it is quite a new field for older adults. Although the use of computers and tablets is steadily rising among older adults in the Netherlands, older adults are limited in adopting new technologies (14). Many questions are still open, such as whether older adults are capable of learning how to use new technologies and whether they will actually use it independently for physical training when offered by professionals (15). For this reason, the primary aim of this study was to investigate the use and user experience of the ICT-supported self-management program when offered for independent use for older adults living at home. Our secondary aim was to explore whether the intervention improved quality of life and health status of these older adults, compared to those in a control group.

## Materials and Methods

### Study Design and Randomization

The study was part of the European FP7 project PERSSILAA (which stands for Personalised ICT Supported Services for Independent Living and Active Ageing). This study was the first wave of a cohort multiple randomized controlled trail (Jansen-Kosterink et al., submitted^1^). This design is introduced by Relton et al. (16) and tackles some of the problems associated with pragmatic trial design. First, a large observational cohort of pre-frail older adults is recruited and their outcomes are measured every 3 months. For each randomized controlled trial pre-frail older adults are randomly selected to the trial intervention. Each person can be randomized only once to an intervention and will stay in this intervention group for the remaining period of time. The outcomes of these randomly *selected* older adults (intervention group) are then compared with the outcomes of pre-frail older adults randomly *not selected (control group)*. This process will be repeated each time a new release of the intervention is available and a randomized controlled trial can start. Study design and procedures were approved by the Medical Ethical Committee of Medisch Spectrum at Enschede, the Netherlands, and all participants provided written informed consent in accordance with the Declaration of Helsinki. The enrollment of participants for the first cohort of the RCT in this study started in September 2014 and ended in January 2015.

### Participants and Setting

This study was conducted in the municipality of Enschede, the Netherlands. Participants were recruited through their general practitioner. They send out a questionnaire for self-screening of general health status to all their older adults between 65 and 75 years old. This self-screening instrument contained various validated questionnaires to assess the level of frailty and functional decline on the physical, cognitive, and nutrition domain. Based on this self-screening instrument, participants were marked as robust (no functional decline on any of the domains), pre-frail (functional decline on one or more domains), or frail (severe functional decline on one of the domains and professional help needed). For this study, pre-frail older adults were asked to participate. To assess the physical domain, participants completed the SF-36 items for the physical functioning scale (PF-10) (17). A score of 60 or below on this scale indicated older adults as pre-frail. Next to this, all older adults with a score of 4 on the Groninger Frailty Indicator (GFI) are marked as pre-frail. For more information about this screening procedure, see the study by van Velsen et al. (18).

**Figure 1:**
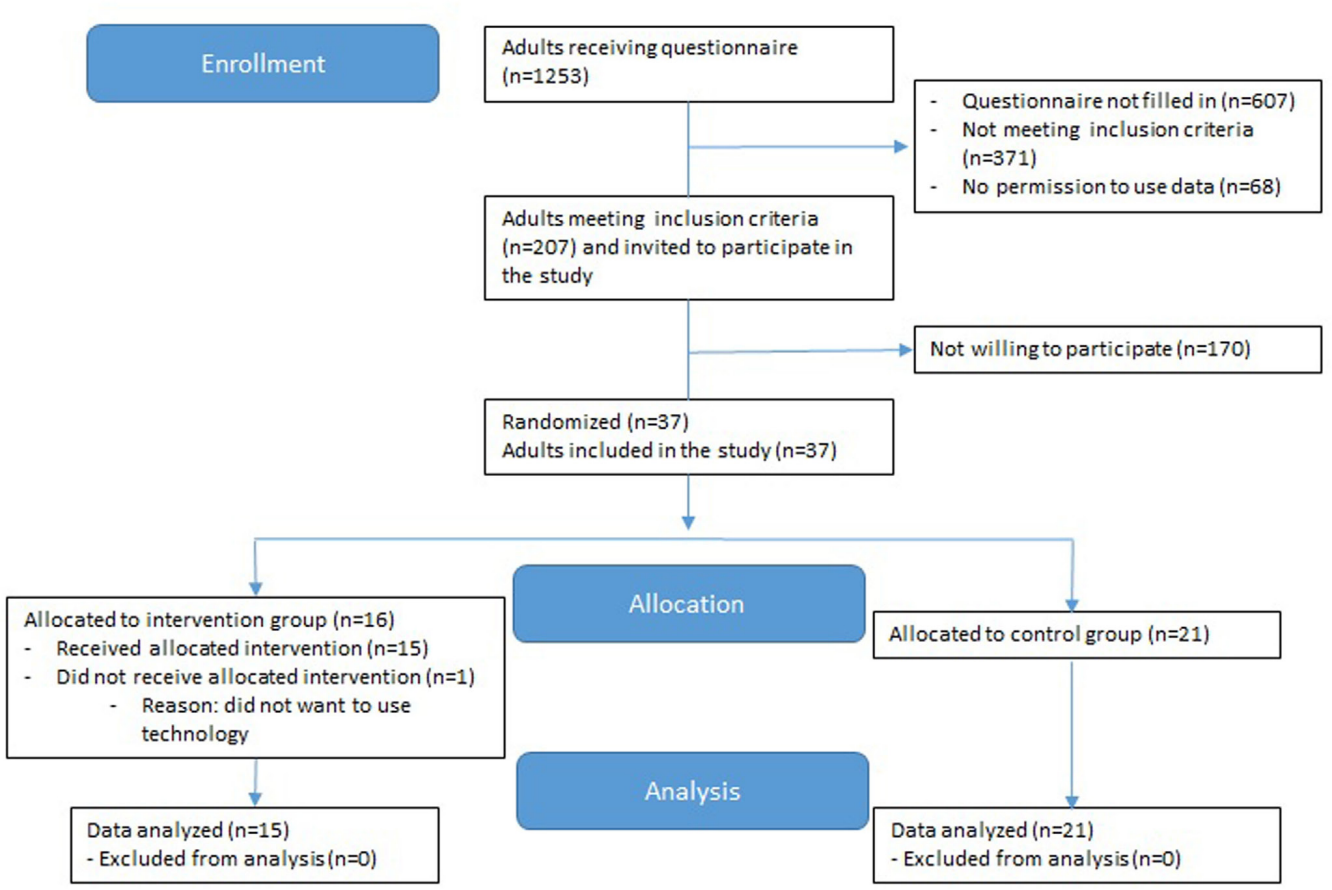
CONSORT flow diagram of progress through the phases of enrollment, allocation, and data analysis.

Participants were excluded if they had conditions that hamper safe execution of the exercise program or insufficient understanding of the Dutch language. Pre-frail participants who were randomly selected to the intervention, but did not fulfill the inclusion criteria for the intervention (such as having a computer with internet at home), remained in the large observational cohort. See Figure 1 for the flow diagram of the study and allocation of participants to the control or intervention group.

### Intervention

**Figure 2:**
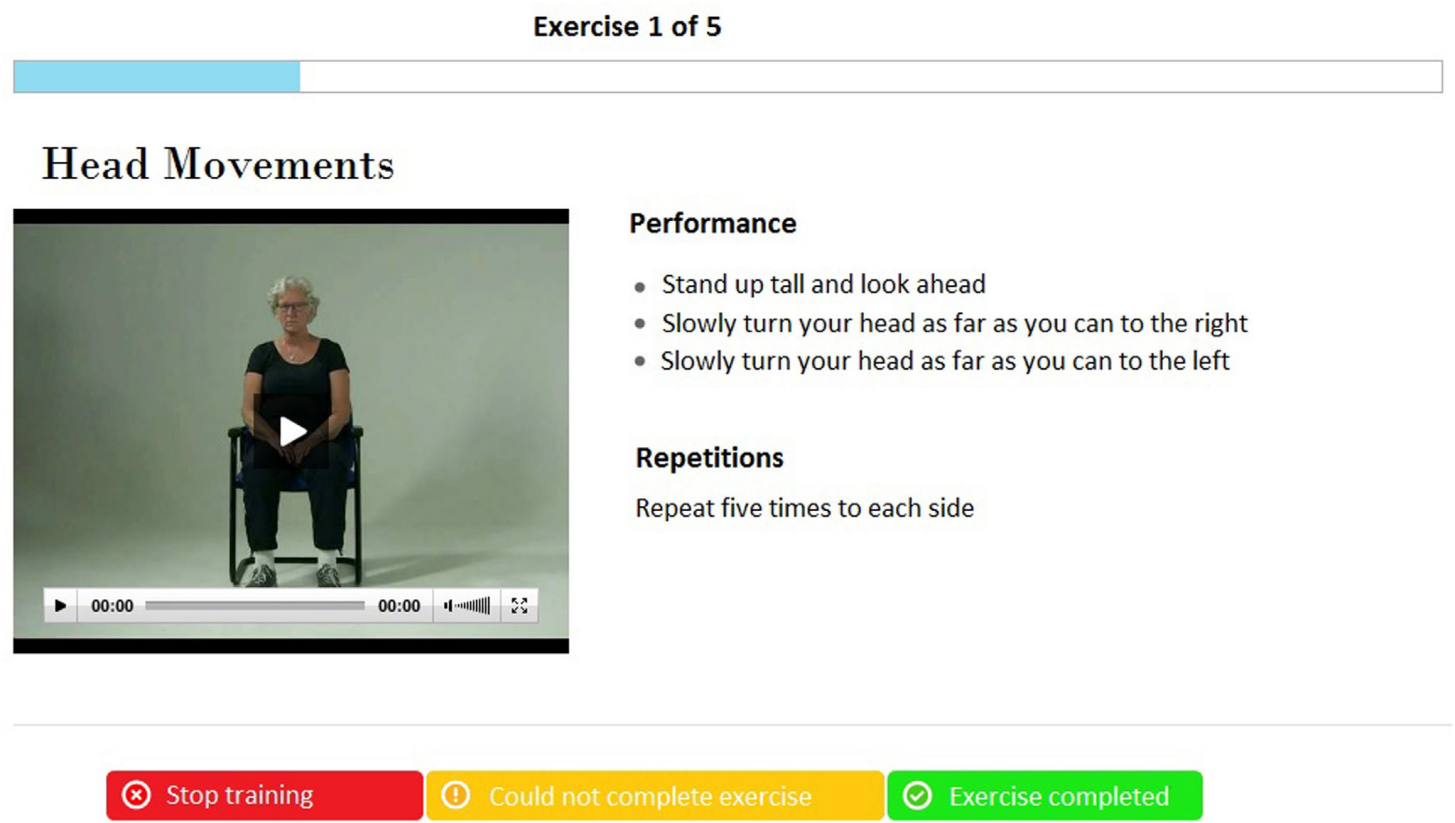
Example of a training video in the exercise program.

The intervention is a technology-supported self-management exercise program, which participants can perform on their own in their home setting. Through a secure login, participants log into a web portal and they can access the program. The home-based exercise program consists of a home-based exercise training that enables participants to train for 3 months. Participants can continue training after program completion. The exercise program is based on the OEP (19), which is an individually tailored fall prevention program used worldwide for muscle strengthening and balance-training exercises of increasing intensity (13). The exercises are functional and closely related to daily activities (e.g., standing up from a chair) and are categorized in three categories: balance, strength, and flexibility. The program consists of 17 exercises each time. Each training session starts with a warming-up of five exercises and ends with a cooling down with the same four exercises. In the training session, participants perform three exercises in each category (balance, strength, and flexibility). Video and step-by-step spoken and written instruction guide the participants through the exercise (see Figure 2).

The use of the program was explained to the intervention group during a plenary meeting.

Participants were advised to take their own computer/tablet with them to this meeting, to make sure the program worked on their computer. During the meeting, participants received their credentials and could start training at home. If they experienced any problems with the program at home, they could call a helpdesk service and someone (SF) answered questions over the phone or would visit the participant.

Participants were advised to train three times a week for approximately 30 min at an individually chosen time, for a duration of 12 weeks. After these 12 weeks, participants could continue using the service for as long as they liked (maintenance phase). No help from physiotherapists is needed to be able to finish the program successfully. However, coaching could be provided when asked for by the participants. When necessary, two physiotherapists could be contacted to support participants with performing exercises at home in a safe manner. When the participant was uncertain about some exercises, he could go to the physiotherapist (exercise point) in a physiotherapy practice in his neighborhood to receive additional feedback. In addition, physiotherapists could log in on the portal with their own secure account, where he has an overview of the training data (frequency of use, difficulties with exercises) of the participants. From this account, they had the possibility to adjust the program to the needs of the participant. The therapist could turn exercises on and off in case they cause any complaints or cannot be performed due to health issues.

The program progresses in four levels. Exercises are randomly and automatically chosen by the program according to the corresponding difficulty level. The first step-in level of the program consists of light and easy exercises, in order to accommodate for sedentary participants. Progression of difficulty in levels elapsed in agreement with the participant. Participants were not obliged to finish all levels during the program. Participants could see their progress in the training module which tells them at what level they are training, which week and which session. After each training session, they got an overview of the exercises performed. The participant had to indicate whether he was able to perform the exercise or not before going to the next exercise. In case he had problems performing the exercise, the question was asked what the problem was. The physiotherapist could view these remarks and adjust the program accordingly, when necessary.

### Control Intervention

The control group had no access to the exercise program and received care as usual with no particular attention, referral, or treatment.

### Data Collection

Questionnaires were assessed before the start of the exercise program (pretest; t0) and after 12 weeks of exercising (posttest; t1).

### Outcome Measures

Demographic and clinical characteristics such as age, sex, years of education, Sf36 physical functioning score, and GFI score will be collected at baseline (t0).

#### Primary Outcomes

The primary outcome measure is use and user experience with the intervention for those participants using the intervention, being measured at t1.

The *use of the intervention* is measured by logging data on the portal and defined in frequency and duration of log in. In addition, we calculated when participants trained during the weeks (morning, afternoon, evening) and which days of the week (weekend versus weekdays).

*Adherence to the program* was calculated according to completion of the training session as indicated by watching the exercise videos. For example, if a participant completed two training sessions in a week, the adherence rate was calculated as 67%.

*User experience* will be assessed by means of the validated System Usability Scale (SUS) (20).

This questionnaire includes 10 items which provide a global view of subjective assessment of system’s usability. SUS scores have a range of 0–100. The higher the scores on the SUS, the better the acceptation of the service. A SUS score above a 68 would be considered above average and anything below 68 is below average. In addition, participants rated their satisfaction on a scale from 1 to 10, with 10 meaning most satisfied.

#### Secondary Outcomes

Secondary outcomes are quality of life and health status. Both will be assessed in the intervention and control group at t0 and t1.

Quality of life is measured with the 12-item Short Form questionnaire version 1 (SF-12v1) (21). The SF-12 is a generic instrument including 12 items measuring health-related quality of life (HRQoL). Six items are summed into a physical component summary (PCS) from the four domains of general health, physical function, physical role limitation, and bodily pain, and six items are summed into a mental component summary (MCS) including the four domains of role limitation, vitality, social function, and mental health. Summary scores are calculated using standard (U.S.) scoring algorithms (22). The total score for both scales ranges from 0 to 100, with a higher number indicating higher HRQoL.

Health status is measured with the EQ-5D-3L questionnaire which consists of five questions covering the following health domains: mobility, self-care, usual activity, pain, and anxiety/depression (23). Each dimension has three possible levels (i.e., 1, 2, or 3), representing “no problems,” “some problems,” and “extreme problems,” respectively. Respondents are asked to choose one level that reflects their “own health state today” for each of the five dimensions. Once the data have been collected and a database created, a scoring function is used to assign a value (i.e., EQ-5D™ index score) to self-reported health states from a set of population-based preference weights. For the Dutch population, the possible EQ-5D™ index scores range from −0.329 (i.e., 33,333) to 1.0 (i.e., 11,111) on a scale where 0.0 = death and 1.0 = perfect health.

The second part is a 20-cm visual analog scale (EQ-VAS) that has endpoints labeled “best imaginable health state” and “worst imaginable health state” anchored at 100 and 0, respectively. Participants are asked to indicate how they rate their own health by drawing a line from an anchor box to that point on the EQ-VAS which best represents their own health on that day.

### Sample Size Calculation

A power calculation based on *t*-test testing shows that at least 40 patients per group are needed (two-sided; SD 8.76; clinical relevant difference in quality of life was set on 5,5 on the SF-12 (24); alpha set at 0.05 and beta 0.2) The SD of 8.76 is derived from a study to the normative data of the SF-12 health survey (25).

### Statistical Analysis

All statistical tests were performed using SPSS (SPSS 17, IBM, New York, NY, USA, version 17). The significance level was set to 0.05. Descriptive characteristics of the participants and baseline values of screening measures are reported as mean with SDs for continuous variables and frequencies for categorical data. A trend is defined as a *p* value < 0.10.

Data were checked for normality using the Shapiro–Wilk test. Paired *t*-tests (normal distributed data) or Wilcoxon-sign rank test (not normal distributed data) were used to evaluate any changes in outcome measures pre- to post-exercise and the demographic differences between the participants in the control and intervention group.

A general linear model for repeated measurements was used to analyze the effects of the intervention on the variables health status and quality of life, with time (pre and posttest) as within and intervention (control or intervention) as between factor. Data were analyzed using the intention-to-treat principle. Wilks’ lambda was used to determine the statistical significance of the multivariate model. Partial eta squared was calculated to measure the effect size.

## Results

Thirty-seven independently living older adults participated in the study. Sixteen participants were randomly allocated to the intervention group. One of these participants dropped out of the study before the study started, because she didn’t want to use technology. Twenty participants were randomly allocated to the control group and one participant didn’t have a computer with internet at home and was automatically allocated to the control group.

### Participants’ Characteristics

Table 1 shows the characteristics of the participants included in the study. No considerable differences between both groups were noted in the baseline data for demographic and clinical measures.

**Table 1:** Demographic and clinical characteristics.

Variables	Control (*n* = 21)	Intervention (*n* = 15)	*p*
Mean age (years, SD) [range min–max]	70,9 (3,5) [66–76]	69,2 (3,8) [65–77]	0.163
Sex	12 females	10 females	0.471
	9 males	5 males	
Highest education (*n*)			0.193
Elementary school	3	2	
High school	5	6	
Lower vocation school	4	–	
Vocational school	2	4	
College	1	2	
Other	5	1	
GFI score	2.9 (0.9)	3.0 (1.3)	0.692
SF36 pf score	33 (18)	43 (17)	0.105

### User Experience

**Figure 3:**
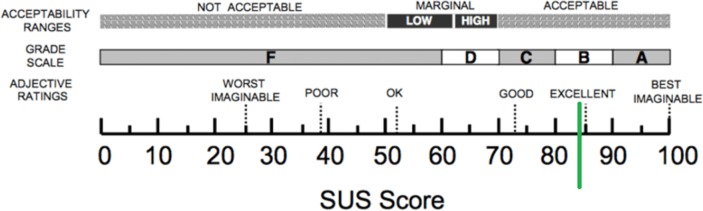
Outcome of the System Usability Scale (SUS).

The average score on the SUS was 84.2 (±13.3) (*n* = 13), which is above average and almost reaching an excellent score (see Figure 3). Participants rated the intervention with an 8.5 (±1.0).

Problems with starting the exercise program in the home environment where mainly related to old internet browsers or no flash player being installed on the computer, which is necessary to be able to watch the exercise movies. These problems have been resolved by the helpdesk.

### Use of the Intervention

Fifteen participants started using the intervention and 12 of them (80%) finished the 12-week exercise protocol. There were three drop-outs (20%) during the study. Two participants stopped using the program, because of health problems (not related to the program) and one participant dropped out because his computer didn’t work during the time of the study. Of the ones finishing the exercise protocol, 11 participants (92%) continued using the service for another 6–40 weeks [mean 25 weeks (± 16)].

Participants preferred to perform the exercises in the morning (43%) and afternoon (47%) compared to the evening (10%). Most of the training sessions were performed during weekdays (83%) compared to training sessions performed during weekend days (17%).

Three participants visited the exercise point weekly (one time a week) to receive some additional feedback about the exercises. For one participant, the therapist used the option to turn some exercises off due to (not program related) health problems (pain in shoulder). This way, this participant was able to continue using the program.

### Intensity and Frequency of Training

**Figure 4:**
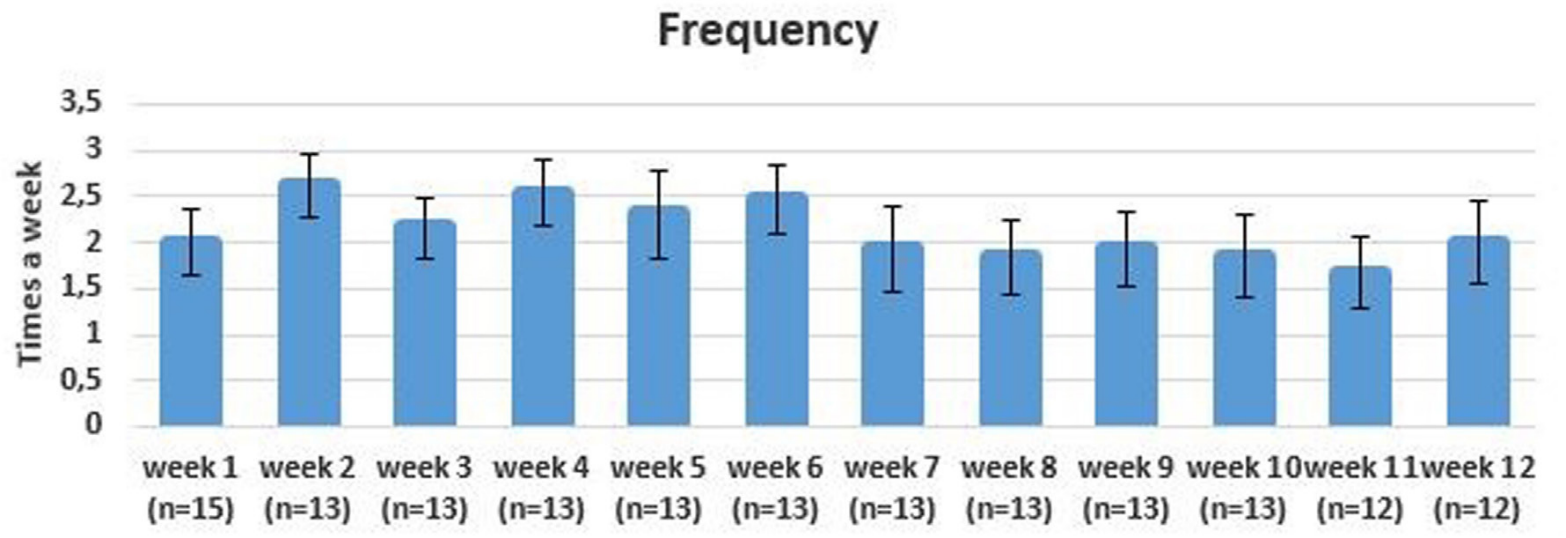
Mean frequency of training each week with error bars.

**Figure 5:**
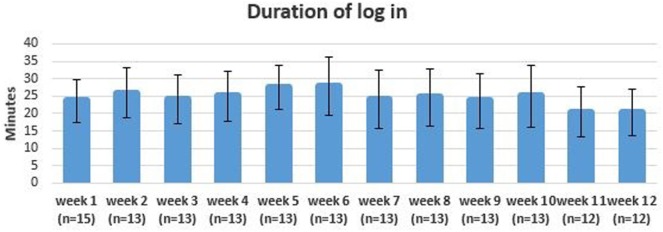
Mean duration of log in each time with error bars.

The adherence to the 3-day exercise protocol was 68%. Participants in the intervention group trained on average 2.2 times (±1.3) each week. The frequency of training decreased during the intervention period with a mean frequency of 2.3 (±1.4) in the first 6 weeks and 1.9 (±1.5) in the last 6 weeks (see Figure 4). The mean duration of login for each exercise session was 24 min (± 8.0) (see Figure 5).

### Quality of Life and Health Status

Table 2 presents the results of the quality of life and health status scores. The intervention group reported greater increases over time on the MCS compared to the control group (*p* = 0.016; partial eta squared 0.178; observed power 0.694). Scores on the MCS of the SF12 were significantly higher in the intervention group pre- and posttest, whereas in the control group there were no significant changes over time. A trend was seen in the change over time in the health status (EQ5D-Index values) (*p* = 0.076). No significant differences between groups were found for the EQ-5D vas scores and the PCS of the SF12.

**Table 2:** Quality of life and health status scores.

Variables	Group	Baseline (t0)	After intervention (t1)	*p*^a^	Time × group effect^b^	Effect size^c^	Power^d^
EQ-5D-3L Index values	Control group (*n* = 18)	0.72 (0.19)	0.70 (0.20)	0.413	0.076	0.098	0.429
	Intervention group (*n* = 15)	0.73 (0.21)	0.79 (0.10)	0.173			

EQ-5D Vas	Control group (*n* = 19)	66.3 (13.3)	64.7 (16.8)	0.849	0.925	0.000	0.051
	Intervention group (*n* = 15)	67.1 (15.3)	65.9 (15.8)	0.488			

SF12 PCS	Control group (*n* = 18)	31.2 (7.3)	30.9 (7.5)	0.873	0.469	0.018	0.110
	Intervention group (*n* = 14)	35.5 (12.5)	33.4 (10.7)	0.293			

SF12 MCS	Control group (*n* = 18)	54.6 (7.4)	54.1 (8.9)	0.486	0.016	0.178	0.694
	Intervention group (*n* = 14)	51.1 (10.3)	56.0 (7.7)	0.017			

*Values representing mean scores (SD)*.

*^a^p-values from paired t-test comparing the effect of the intervention on pre- and posttest outcome parameters*.

*^b^p-value from GLM*.

*^c^Effect size partial eta squared from GLM*.

*^d^Observed power from GLM*.

## Discussion

The objective of this study was to investigate the use and user experience of an online home-based exercise program and to determine whether the intervention improved quality of life and health status of physically pre-frail older adults compared to a control group.

In our study, older adults were advised to train three times a week for a period of 12 weeks. The adherence to this protocol was 68%. The drop-out rate was low (20%) and participants trained on average two times a week. We are satisfied with this adherence rate, as different studies showed that strength training on two nonconsecutive days per week may be as effective as more frequent exercise sessions in older adults (26, 27). However, the frequency of training decreased over time with on average less than two training sessions in the last 6 weeks. Adherence to self-managed programs in older adults is an important issue in other studies as well (28, 29) and adherence rates are generally higher in supervised programs compared to non-supervised programs (30, 31). This might reduce the effectiveness of self-management exercise programs. However, current developments in technology enable different strategies to assist in improving adherence to home-based exercise programs for older adults with minimal supervision. Different theories show that motivational strategies are important in enhancing adherence to treatment and successful motivational interventions confirm the persuasiveness of personal, individualized feedback (32). Home-based exercise programs provide an opportunity to tailor exercises to the individual needs in contrast to many group-based physical activity programs. This is an advantage that can be more elaborated in the program. Different individual parameters could be taken into account by using for example recommender techniques. Smart algorithms can be used in order to define an appropriate and more encouraging exercise program for the participant, by taking into account the exercises the participant likes, time of the day, age, and gender (33). Future research should focus on the effectiveness of these specific motivation strategies in order to increase exercise adherence for self-management programs for older adults.

Our study shows that most of the older adults were very enthusiastic about the service and didn’t experience problems with the new technology when being trained beforehand. Overall, the program is well accepted and the participants are very satisfied with the program. Only three participants (20%) visited the exercise point to receive feedback about the exercises and for only one participant it was necessary to adjust the exercise program. The majority of older adults experienced no problems with the technology, which creates a future for the use of self-management interventions for older adults. A helpdesk, training before the start of the study and an exercise point during the study were helpful in our study to decrease the amount of technical and health problems. This suggests that it is important to implement such new technologies as a service and not as a stand-alone-program. Vollenbroek-Hutten et al. (34) also showed that it is important to implement new ICT services *via* a neighborhood facility instead of directly totally independent at home.

The secondary research question was whether the new intervention improves the quality of life and health status of pre-frail older adults. There is a positive result regarding HRQoL, which shows significant improvements between groups on the mental scale of the SF12. In addition, a trend was seen in improvement of health status between groups. These results are in line with a recent review investigating the aging process, which showed that health promotion interventions are beneficial in terms of improvement in quality of life and/or physical well-being (35). Although positive results, further research is necessary to investigate whether our results are related to adherence measures and if these effects hold over time compared to the control group. This would provide insight whether such ICT-supported self-management programs have potential in preventing and/or delaying the frailty process.

### Limitations

Several limitations need to be taken into consideration when interpreting our findings. The small sample size in our study was limited and as such the clinical findings need to be interpreted with caution. More research is necessary to understand the value of the results found in this study. Another limitation was that testers were not blinded to group assignment for ease of recruitment and to accommodate for time constraints. Blinding, however, is a critical methodological feature of a randomized control trial. This needs to be taken into consideration when evaluating the validity of the results.

## Conclusion

This study provides evidence that a home-based exercise program is easy to use and has potential in improving quality of life and health status of physically pre-frail, older adults who live at home. However, further refinement of the program is required to improve adherence and maximize the benefits and potential of exercising in the home environment.

## Ethics Statement

This study was approved by the Medical Ethical Committee of Medisch Spectrum at Enschede, the Netherlands. All participants gave written informed consent in accordance with the Declaration of Helsinki.

## Author Contributions

All authors participated in writing the manuscript, revising it critically for important intellectual content, and gave their final approval for publishing the manuscript.

## Conflict of Interest Statement

The authors declare that the research was conducted in the absence of any commercial or financial relationships that could be construed as a potential conflict of interest.

## Footnotes

^1^Jansen-Kosterink S, Vollenbroek-Hutten MMR. The evaluation of an online platform for older adults to monitor and train their physical and mental capacity and develop proper eating habits: a study protocol of a cohort multiplerandomized controlled trial. Submitted to *BMC Geriatrics* (2017).
